# Single-Incision Laparoscopic Cholecystectomy: Is It a Plausible Alternative to the Traditional Four-Port Laparoscopic Approach?

**DOI:** 10.1155/2012/347607

**Published:** 2012-05-09

**Authors:** Juan Pablo Arroyo, Luis A. Martín-del-Campo, Gonzalo Torres-Villalobos

**Affiliations:** ^1^Molecular Physiology Unit, Instituto de Investigaciones Biomédicas, Universidad Nacional Autónoma de México and Instituto Nacional de Ciencias Médicas y Nutrición Salvador Zubirán, Zubirán, 14000 Mexico, df, Mexico; ^2^Surgery Department, Instituto Nacional de Ciencias Médicas y Nutrición Salvador Zubirán, Zubirán, 14000 Mexico, DF, Mexico; ^3^Experimental Surgery Department, Instituto Nacional de Ciencias Médicas y Nutrición Salvador Zubirán, Zubirán, 14000 Mexico, DF, Mexico

## Abstract

The current standard-of-care for treatment of cholecystectomy is the four port laparoscopic approach. The development of single incision/laparoendoscopic single site surgery (SILC/LESS) has now led to the development of new techniques for removal of the gallbladder. The use of SILC/LESS is now currently being evaluated as the next step in treatment of cholecystectomy. This review is an attempt to consolidate the current knowledge and analyze the feasibility of world-wide implementation of SILC/LESS.

## 1. Introduction

The ultimate goal of surgery has always been providing the best and most effective procedure with the least amount of postoperative complications, and pain and the best possible aesthetic results. Surgery of the biliary tract is by no means the exception. The first reported elective cholecystectomy was carried out by Langenbuch in 1882 [1] and open cholecystectomy became the standard-of-care well into the 1980s with mortality rates at less than 1%, and bile duct injuries affecting 0.1-0.2% of patients [2, 3]. This approach however required a large abdominal incision associated with significant postoperative pain and a longer convalescence.

A revolution in the surgical treatment of biliary disease came in the 1980s with the introduction of laparoscopic surgery. The first laparoscopic cholecystectomy was performed by Mühe [4] however his approach did not become popular until both French and American groups popularized the four-port technique in the early 1990s. The idea of minimally invasive surgery for the removal of the gallbladder had now become a plausible technique that was rapidly accepted as the standard-of-care. Patients quickly learned of the new procedure and began to request it on the basis of a shorter hospital stay, less pain, and smaller scars [5]. The possibility of performing laparoscopic cholangiography, common bile duct exploration, and choledochotomy expanded the role of laparoscopic surgery in the treatment of biliary disease [6] and further advanced the idea of minimally invasive surgery as the gold-standard for surgery of the biliary tract.

Recently the development of natural orifice transluminal endoscopic surgery (NOTES) opened the field of incision-less surgery. The main goal of NOTES is to eliminate the need for skin incisions along with other theoretical advantages which include: decreased postoperative pain, performing procedures in the out-patient setting, reduced incidence of hernias, reduced hospital stay, and increased overall patient satisfaction [5, 7]. The idea of accessing internal organs through the wall of the vagina, colon, stomach, bladder, and so forth, with the use of rigid or flexible instruments is an attractive one. However, the challenge of obtaining a clean access site thereby preventing intra-abdominal spillage or infection from the incision has not been able to be fully avoided [7]. Additionally the concern over closure of the luminal incision and the lack of a single effective closure technique for stomach, esophagus, or colon, so far limits the application of this technique. Moreover, the possibility of generating bowel-overdistention due to the pneumoperitoneum required for adequate visualization of intra-abdominal structures is still a concern [5]. With current ongoing research on the efficacy and safety of NOTES it is still premature to advocate it as an alternative to laparoscopic surgery of the biliary tract.

Single-incision laparoscopic surgery or SILS refers to the operative technique in which a surgical procedure is carried out through one incision, alternatively it is also known as laparoendoscopic single site (LESS) surgery. In 1997 Navarra et al. described a single-incision laparoscopic cholecystectomy as a plausible alternative procedure to the four-port laparoscopic cholecystectomy [8]. The use of a single umbilical incision to remove the gallbladder was an interesting innovation and, since Navarra's initial description, the single-incision laparoscopic cholecystectomy (SILC) procedure has gained momentum. The goals of SILC/LESS cholecystectomy are similar to the goals behind the development of NOTES: decreased pain, decreased length of hospital stay, better aesthetic results, and increased patient satisfaction among others [6, 9]. Multiple articles regarding the use of SILC/LESS cholecystectomy have been published since the initial two studies were published by Bresadola et al. [10] and Piskun and Rajpal [11], leading to a wealth of information regarding the possible adoption of the SILC/LESS cholecystectomy by surgeons worldwide, including a 2010 consensus statement by the Laparoendoscopic Single-Site Surgery Consortium for Assessment and Research (LESSCAR) [9]. It is our goal to review the different SILC/LESS cholecystectomy techniques reported so far along with the results associated with the most recent SILC/LESS cholecystectomy trials.

## 2. Technical Aspects of Laparoendoscopic Single Site Cholecystectomy

Due to the growing experience and development of ports and instrumentation, surgical technique for LESS cholecystectomy is rapidly evolving [21]. A particular technical challenge for the LESS approach is limited triangulation due to confinement of both optics and working instruments to a single axis. Researchers and the industry are pursuing solutions to this through the development of next-generation instruments (Angled, flexible, articulated, and motorized) [9].

Given this, there is a wide variation of methods regarding the type of ports, trocars, optics, instruments, and methods to expose and dissect the gallbladder (Table 1). Nevertheless, many LESS procedures (including cholecystectomy) have been successfully performed with conventional laparoscopic instruments.

### 2.1. Surgical Technique

#### 2.1.1. Patient Position

The patient is placed in supine or the split-leg position, with the surgeon standing on the patient's left [22] or between the patient's legs [23]. According to the surgeon's position, the assistant is placed either on the patient's right or left. After access to the abdominal cavity is obtained, the patient will be placed in reverse Trendelenburg with a slight rotation to the left to clear abdominal organs from the gallbladder [24].

#### 2.1.2. Abdominal Cavity Access

Access can be accomplished by two approaches [25]:

- LESS devices (Table 1) are designed to deploy through a single incision (typically at the umbilicus) and require a fascial incision of approximately 15 to 25 mm [14];
- single incision with multiple trocars uses commercially available laparoscopic ports placed through a single incision with a bridge of fascia between them [26]. A particular concern about this approach is the risk for increased hernia rates given the unknown effect of multiple fascial punctures in proximity [25], although to this date, there are no reports of different hernia rates between these two approaches.

#### 2.1.3. Gallbladder Exposure

Most of the initial experience in LESS cholecystectomy relies on gallbladder suspension using transparietal stitches [6, 27]. Although different approaches have been described, the principle is to place one to three stitches in the gallbladder fundus and/or infundibulum and apply different degrees of tension to expose the Calot's triangle while using another instrument to dissect [28].

Nevertheless, some authors advocate for abandoning transparietal stitches for exposure, as they may be associated with accidental puncture and a potential oncological risk [21]; therefore, they prefer an intracorporeal grasper placed through a transumbilical port or a SILS port to gain dynamic exposure. Also, the use of an additional 1.8 to 3 mm grasper introduced through the skin has been used to assist cephalad retraction and has not been considered as conversion in recent clinical trials [18, 19]. There is also a report of extracorporeal retraction using magnet forceps attached to the gallbladder [29].

#### 2.1.4. Calot's Triangle Dissection

One should always consider that a less invasive procedure must also be safe. Therefore, every effort must be made to comply with the requirements of the critical view of safety for laparoendoscopic cholecystectomy [30], that comprises dissection of the neck of gallbladder off the liver bed to achieve conclusive identification of the two structures to be divided: the cystic duct and the artery.

Instruments used for this purpose are very similar to those of 4-port laparoscopic cholecystectomy and include 5 mm hook, dissector scissor, and angle dissector. The cystic duct and artery are then dissected free, secured with clips, and divided [22].

#### 2.1.5. Gallbladder Bed Dissection

Although gallbladder dissection can be accomplished with a fundus-first technique [19], we encourage to do it after preparation of the cystic duct and artery (Strasberg critical view). Dissection is usually performed with a hook type electrocautery device [24].

#### 2.1.6. Extraction

After cholecystectomy has been completed, the gallbladder can be extracted through the LESS port, as it acts as a wound protector [17], or using a specimen bag that is introduced through the umbilical port when traditional laparoscopic instruments are being used. When using laparoscopic instruments, extraction through 5 mm ports is unfeasible and they will need to be increased to 10 or 12 mm [6].

#### 2.1.7. Wound Closure

The fascial incision is closed with a figure of eight stitch [18]. Deep dermis of the umbilicus is reapproximated to ensure cosmesis [23].

### 2.2. Current Application

The current status of single-site surgery poses several technical difficulties for the surgeon [9], and cholecystectomy has not been the exception. Current consensus recommends that LESS procedures are only performed in centers with adequate laparoscopic experience and by surgeons with a certain amount of LESS surgical training [9].

Nevertheless, Mutter et al. have shown that LESS cholecystectomy can be safely implemented in a teaching hospital with both senior and junior laparoscopic surgeons [31]. For surgeons that are proficient with multi-incision laparoscopic cholecystectomy, the learning curve for LESS cholecystectomy begins near proficiency with infrequent complications and conversion rates [32].

### 2.3. Technical Strategies

In order to overcome the limitations of triangulation with the LESS approach, several approaches have been proposed. Curved and or articulated instruments have been used according to the surgeon's preference [14], as they may allow to work on the operative field without a straight approach from the access port. Using these instruments requires the instrument from the right hand to be on the left side of the screen and the left-hand instrument to be on the right side of the screen [6, 33].

One can choose an instrument with handles that are articulated so they are away from each other at the access port or use ports with a lower external or internal profile for a wider range of instrument motion. Also, instruments of variable lengths allow for external manipulation so that they are operated in different planes, thus avoiding collisions [25].

## 3. Patient Outcomes: SILC/LESS cholecystectomy versus Four-Port Cholecystectomy

In spite of numerous reports regarding the safety and efficacy of the SILS/LESS cholecystectomy approach, laparoscopic cholecystectomy (LC) still remains the gold-standard for the surgical removal of the gallbladder [6]. Thus the comparison of patient outcomes between both procedures is of key importance. In this respect several prospective studies comparing LC and SILC/LESS Cholecystectomy have now been published [12–20] (Table 2).

There are several blinded randomized trials comparing standard LC to SILC/LESS cholecystectomy with varied results regarding patient outcomes. An outcome that has had a significant difference in several studies comparing SILC/LESS cholecystectomy versus LC is the cosmetic result. Patients are more satisfied with the hidden or infraumbilical single surgical scar than the four scars created by the LC [13, 17, 19]. In an attempt to try and reduce the bias associated with cosmetic evaluation, Marks et al. and Bucher et al. used body image scale, a scar scale photo series 10-point scoring questionnaire in order to compare results between SILC/LESS and LC patients. However regardless of the scale used, there is still an element of personal preference and opinion involved with the evaluation of cosmetic results.

Aside from cosmetic perception, the only consistently reproducible and statistically significant result among series is a prolonged time of surgery for the SILC/LESS cholecystectomy groups versus standard LC groups [12–14, 16–20]. A study by Qiu et al. [34] focused specifically on the learning curve phenomenon associated with SILC/LESS cholecystectomy and saw an improvement in operative times as experience was gained [34] this was similar to what was observed by others [18–20]. The increased operating time may be a combination of factors among which the lack of surgeon experience and the technical difficulty behind SILC/LESS cholecystectomy could be involved. However, increased operating time means increased duration of general anesthesia and thus increased patient risk. Although no anesthesia-related complications were reported in the mentioned trials, a significant number of the studies used ASA class III or IV as a cut-off point for patients suitable for SILC/LESS cholecystectomy [13, 14, 19], thus the use of SILC/LESS cholecystectomy in patients in which there are foreseeable anesthesia-related complications remains limited.

One of the ultimate goals of the development of SILS/LESS cholecystectomy is a reduction in postoperative pain perception and a decreased used of analgesic medications [9]. The evaluation of postoperative pain is consistently included as a primary or secondary outcome in recent studies [12–20] but lacking in previous studies [6]. The outcome however remains obscure as there are reports in which there is no difference in pain perception between SILC/LESS cholecystectomy and LC groups [14, 16, 18], increased perception in the SILC/LESS cholecystectomy group [15, 19], and decreased pain perception in the SILC/LESS cholecystectomy groups [12, 17]. The lack of consistent evidence regarding pain perception requires further evaluation in randomized clinical trials.

In comparing outcomes between procedures, one of the key points to evaluate is the presence or absence of intraoperative and postoperative complications. A procedure can be considered safe only if the rate of complications is similar to that of the current gold-standard. When comparing the rate of complications between SILC and LESS cholecystectomy numerous studies have reported both, no significant difference with regard to complication rate [6, 15, 17, 22] or an increased complication rate when comparing SILS/LESS cholecystectomy to LC [14, 18]. With regard to the study by Phillips et al. [14] it is interesting to note that this is the same cohort of patients as an initial report by Marks et al. In the original report by Marks et al. [13] there was no significant difference in complications. However in the report by Phillips et al. [14], the number of patients increased and so did the complications associated with single-incision surgery [14]. This is the largest case series published so far and in theory the learning curve has leveled off, indicating that the complications are inherent to the procedure itself, questioning the feasibility of widespread application of the SILC/LESS cholecystectomy. One of the complications that has been discussed the most is the increased risk of a postincisional hernia after SILS/LESS surgery due to an increase in size of the defect in the fascia. This complication has tried to be avoided by turning multiple fascial defects into a single incision, however, results have been inconclusive. [6, 14, 25, 35].

Previous data on patient outcomes after SILC/LESS cholecystectomy suggest that this new procedure is reproducible and safe [9], however this does not seem to agree with the results from the recent RCTs (see above). The literature on SILS/LESS cholecystectomy has been recently reviewed by Antoniou et al. [6]. They analyzed the results of 29 different articles reporting the realization of a SILC/LESS cholecystectomy with a total of 1166 patients. Among the reported results there is 9.3% of unsuccessful surgery, generally due to a lack of proper identification of Calot's triangle, along with a cumulative intraoperative complication rate of 2.7% (range 0–20%) with the most common being gallbladder perforation/bile spillage (2.2%) and hemorrhage (0.3%). The most common postoperative complications were wound infection and hematoma in 2.1% of patients [6].

In more recent articles Duron et al. and Mutter et al. reported series of 55 and 58 patients, respectively, who underwent SILC/LESS Cholecystectomy [31, 36]. Duron et al. [36] reported a series of 55 cases performed in a single institution, in which a “learning curve” effect was present with regard to shorter operating times and the inclusion of more technically difficult patients as surgeon experience increased [36]. Mutter et al. [31] analyzed the implementation of this type of surgery in a teaching hospital comparing six surgeons (3 senior surgeons and 3 junior surgeons) finding no significant difference between operating times or complication rates, thus advocating the safe implementation of SILC/LESS cholecystectomy in teaching hospitals [31]. These results however, include a limited number of surgeons and are applicable only to patients with programmed cholecystectomies without any foreseeable factors aggravating dissection of Calot's triangle as out of the 58 patients only 3 were diagnosed with acute cholecystitis, thereby limiting their applicability.

In a matched pair analysis that took place over 26 months, Gangl et al. [20] compared operating time, postoperative pain using the visual analogous scale (VAS) at 24 and 48 hrs, use of analgesics, length of hospital stay, and complications [20]. They performed the SILC/LESS patient data gathering prospectively, comparing them to matched controls from a group of 163 LC which were performed in the same time period, with no significant differences in age, gender, BMI, ASA classification, diagnosis of acute cholecystitis, or previous abdominal surgery. They reported a SILC/LESS cholecystectomy completion rate of 85.1%, with conversion to LC in 9 patients and open cholecystectomy in 1 patient due to inadequate visualization of the anatomy, versus a 100% completion rate in the LC group, with no significant difference with regard to postoperative pain, analgesic use, length of stay or complications. The only significant difference was the length of surgery with a longer operating time in the SILC/LESS cholecystectomy group (75 min versus 63 min). They conclude that SILC/LESS even though associated with a longer operating time is comparable to LC [20].

The incidence of biliary injury during standard LC varies from 0.5 to 0.8% [37]. In order to identify biliary injury the use of intraoperative cholangiogram is now considered a standard procedure to evaluate anatomy of the biliary tree. The possibility of carrying out a transoperative cholangiogram in SILC/LESS was recently evaluated by Yeo et al. [38]. They were able to observe that in the 55 patients in which a successful SILC was carried out, 53 received a transoperative cholangiogram out of which 48 were normal with 1 patient requiring endoscopic removal of a biliary stone [38]. This is the largest series of SILC/LESS which reports the routine evaluation of biliary anatomy with a cholangiogram performed through an umbilical port, however, whether these results are reproducible or not, requires further studies. A more pressing issue regarding biliary injury and SILC/LESS is an adequate exposure of Calot's triangle or “the Strasberg critical view.” As described above, in order to achieve the “critical view,” the use of transparietal sutures or magnetic forceps that allow extra corporeal traction on the gallbladder fundus can be carried out [6, 21, 29]. It is interesting to note that in the study carried out by Antoniou et al. [6], the two most common reasons for conversion from SILC/LESS to standard LC were: Inflammation/adhesions/unclear anatomy (47.4% of all conversions) and inadequate visualization of Calot's triangle (23.7% of all conversions) with a total rate of 5.2% and 2.6%, respectively [6]. The lack of an adequate identification of the anatomical landmarks be it by inflammation, adhesions, or normal anatomical variants is worrisome due to the increased incidence of bile duct injuries in the presence of a less than adequate exposure [39].

When comparing costs, the cost of SILS/LESS cholecystectomy was increased compared with that of LC in spite of the authors in the Bucher et al. [21] study reutilized as much material as possible. They hypothesized that the costs are a reflection of product development, and that as of now costs are not comparable to those of a routine procedure such as LC [17]. In contrast, a study by Love et al. [40] in which cost comparison between 20 patients undergoing each procedure did not yield a significant cost difference [40]. Thus the issue of comparing cost is far from over, particularly if there are still a myriad of technical options available for the realization of a SILC/LESS cholecystectomy and there is no standardized instrumentation.

## 4. Conclusions

Current evidence suggests that even though patients prefer the cosmetic result of SILC/LESS cholecystectomy over a traditional laparoscopic approach [41], SILC/LESS cholecystectomy is still a long way off from replacing laparoscopic cholecystectomy as the gold-standard for surgical removal of the gallbladder. Insufficient evidence regarding the safety, complication rate, and costs seems to preclude the worldwide implementation of this minimally invasive procedure. Additional concerns exist regarding patient safety if it is not a programmed surgery, thus rendering SILC/LESS cholecystectomy unavailable to a large subset of patients. Initial data showing increased complication rate along with a longer operating time, lack of standardization, and instrumentation makes SILC/LESS cholecystectomy still an experimental procedure that requires further development in order to be applicable to general surgeons worldwide.

## Figures and Tables

**Table 1:** Commercially available multiport systems.

Port system	Manufacturer
AnchorPort	Surgiquest Inc (Orange, CT, USA)
GelPOINT	Applied Medical (Rancho Santa Margarita, CA, USA)
SILS Port	Covidien (Norwalk, CT, USA)
TriPort	Advanced Surgical Concepts (Wicklow, Ireland)
Uni-X Single Port	Pnavel Systems (Brooklyn, NY, USA)

**Table 2:** Comparison of clinical trials comparing SILC versus 4PLC—SILC/LESSC (single-incision laparoscopic cholecystectomy/laparoendoscopic single-site cholecystectomy), 4PLC (four port laparoscopic cholecystectomy).

Study	Study type	Year	No. of patients	Inclusion criteria	Exclusion criteria	Primary outcomes	Secondary outcomes	Mean operative	
								time (min)	
								SILC/LESSC	LC
Tsimoyiannis et al. [12]	Prospective, randomized	2010	40	BMI < 30, pain from cholelithiasis, ASA class I or II	BMI > 30, acute cholecystitis, choledocholithiasis or acute pancreatitis	Postoperative pain* (less pain in SILSC group)	NR	49.65 ± 9.02*	37.3 ± 9.16

Marks et al. and Phillips et al. (same cohort of patients) [13, 14]	Prospective, randomized,	2011	200	BMI < 45, diagnosis of biliary colic, with gallstones or polyps, biliary dyskinesia EF < 30%.	Pregnancy, acute cholecystitis, preoperative indication for cholangiogram, ASA class III or IV, peritoneal dialysis, umbilical hernia	Intraoperative, postoperative complications (up to 1 yr)*, operative time*, and estimated blood loss.	Pain evaluation* (less pain in 4PLC group), cosmesis*, quality of life, time required for insertion of SILS/LESSC port versus LC ports	57.2*	45.2

Lai et al. [15]	Prospective, randomized,	2011	51	Age 18–80 yrs, diagnosis of symptomatic gallstones or polyps scheduled for elective cholecystectomy	ASA class IV or V, contraindication to laparoscopy, the Mirizzi syndrome, suspected common duct stones, suspected malignancy, previous upper abdominal surgery, long-term anticoagulation, previous history of cholangitis/cholecystitis, gallstones >3 cm, contracted gallbladder or chronic cholecystitis	Postoperative pain* (less pain in LC group)	Open conversion rate, surgical complications, hospital stay, resumption of normal life, cosmesis	43.5 ± 15.4	46.5 ± 20.1

Lee et al. [16]^+^	Prospective, randomized	2010	70	Symptomatic colelithiasis, ASA class I or II	Acute cholecystitis, common bile duct stones, severe obesity and previous upper abdominal surgery	Postoperative pain	Duration of surgery, complications, analgesic requirements, length of hospital stay*, cosmesis*, wound length*, time to return to work	71.7 ± 11.6*	48.4 ± 10.5

Bucher et al. [17]	Prospective, randomized	2011	150	Elective patients with symptomatic gallbladder stones, history of cholecystitis, history of common bile duct stone migration and/or biliary pancreatitis, age > 18 yrs	Acute gallbladder disease, contraindications to pneumoperitoneum, cirrhosis, mental impairment	Cosmesis*	Postoperative pain* (less in SILC/LESSC group), analgesia requirement*, satisfaction*, morbidity, duration of operation, need for main port expansion for specimen retraction*, hospital stay, return to work* and operative costs*	66 (no SD reported)	64 (no SD reported)

Ma et al. [18]	Prospective, randomized	2011	43	Indications for LC with no evidence of choledocholithiasis, age 18–85 yrs, BMI < 40, creatinine < 2 mg/dL, AST/ALT <5x upper limit of lab normal, normal total bilirubin	Acute cholecystitis, gallstones >2.5 cm	Postoperative pain	Operative time, length of hospital stay, postoperative morbidity, QOL, cosmesis	88.5*	44.8

Lirici et al. [19]	Prospective, randomized	2011	40	age 18–75, BMI < 30, no previous abdominal surgery, gallstones on US exam. ASA class I–III, Nassar grade I–III	BMI > 30, previous abdominal surgery, acute cholecystitis, bile duct stones, pancreatitis, ASA class > III, Nassar grade IV	Length of stay, postoperative pain* (higher with SILC/LESSC on the day of surgery, rest NS), cosmetic results*, SF-36 questionnaire scores* (Role Emotional only, rest NS)	Operative time*, conversion to LC, difficulty of exposure*, difficulty to dissect, complication rate	76.75*	48.25

Gang et al. [20]	Prospective, matched pair analysis	2011	134	SILC/LESSC patients matched to LC controls		Completion rate, operating time, postoperative complications, length of stay, postoperative pain		77 ± 26*	68 ± 31

^+^SILC versus mini laparoscopic procedure.

*Statistically significant difference.
